# Effect of Hyperglycemia on Purinergic and Nitrergic Inhibitory Neuromuscular Transmission in the Antrum of the Stomach: Implications for Fast Gastric Emptying

**DOI:** 10.3389/fmed.2018.00001

**Published:** 2018-01-23

**Authors:** Xue-Dao He, Yan-Mei Guo, Raj K. Goyal

**Affiliations:** ^1^Department of Medicine VA Boston Healthcare System, Harvard Medical School, Boston, MA, United States

**Keywords:** fast gastric emptying, slow gastric emptying, vagovagal reflexes, neuromuscular transmission, diabetic stomach

## Abstract

**Background:**

Hyperglycemia has been reported to enhance vagovagal reflex that causes the release of inhibitory neurotransmitter, nitric oxide (NO), at the neuromuscular junction in the antrum to relax the antrum and slow gastric emptying by stimulating glucose-sensitive afferent neurons. However, hyperglycemia has also been reported to cause fast gastric emptying that may be due to suppression of the inhibitory motor neurons.

**Aims:**

The purpose of the present study was to investigate changes in inhibitory neuromuscular transmission in the gastric antrum due to hyperglycemia.

**Methods:**

Inhibitory electrical junction potentials were recorded from gastric antral muscle strips, using intracellular electrodes under non-adrenergic, non-cholinergic conditions. Studies were performed in non-hyperglycemic NOD (NH-NOD), NOD mice as they develop hyperglycemia (H-NOD) and their age-matched controls. The purinergic inhibitory junction potential (pIJP) and nitrergic IJP (nIJP) were isolated pharmacologically.

**Results:**

The control pIJP was large, around −18 mV and nIJP was small, around −9 mV. In NH-NOD the IJPs were not affected, but in H-NOD pIJP was nearly abolished and nIJP was significantly reduced. In H-NOD mice, membrane hyperpolarization caused by exogenous α,β-MeATP or diethylenetriamine NO adduct was similar to that in wild-type controls (*P* > 0.05). H-NOD smooth muscles were significantly depolarized as compared to NH-NOD smooth muscles.

**Conclusion:**

These observations show that hyperglycemia causes suppression of purinergic and nitrergic transmission by acting on the motor neurons that form the last neuron in the vagovagal circuit. Moreover, the loss the neurotransmission is due to a defect in neurotransmitter release rather than a defect in signal transduction. Hyperglycemia also causes depolarization of smooth muscles that may increase their excitability.

## Introduction

Vagus nerve and myenteric plexus play a central role in the regulation of gastric motility and gastric emptying (1). The neural circuits that mediate vagovagal reflexes include vagal afferents with their cell bodies located in the nodose ganglion and their centrally projecting fibers found in the nucleus tractus solitarius, that synapse on neurons in the dorsal motor nucleus of the vagus (DMV). The DMV houses preganglionic neurons for parallel excitatory and inhibitory pathways, that synapse with excitatory and inhibitory postganglionic motor neurons, respectively, located in the myenteric plexus in the stomach. The postganglionic vagal neurons are shared with the myenteric motor neurons and are also connected with other myenteric neurons, including the intrinsic afferent neurons. The myenteric plexus mediates local intragastric reflexes that integrate activities of different parts of the stomach that is required for gastric motility and gastric emptying. The vagal postganglionic/myenteric inhibitory and excitatory neurons are final components of the vagovagal or local myenteric plexus circuits. The main inhibitory neurotransmitters are nitrergic [nitric oxide (NO)], purinergic, and peptidergic (VIP) in nature, and the main excitatory transmitter is cholinergic (2, 3).

Several studies have shown that gastric fundus and proximal corpus constitute the pressure pump and is involved in gastric emptying of liquids and solids and distal corpus and antrum constitute the peristaltic pump and is particularly involved in gastric emptying of solids but also liquid chyme. Pylorus acts as a grinder and regulates gastric emptying of solids (4, 5). Hyperglycemia is considered to delay gastric emptying, both in healthy controls and in patients with diabetes mellitus (6). However, parenteral hyperglycemia has been shown to produce variable effects on gastric emptying rates, depending on the nature of the meal (7). Hyperglycemia-induced slow gastric emptying is due to gastric relaxation and reduced antral contractions (8, 9). Hyperglycemia is reported to cause gastric relaxation and reduced antral contractions induced by insulin hypoglycemia by a central action on the nuclei of vagal afferents (10).

The vagovagal reflex that induces gastric relaxation and slow gastric emptying involve stimulation of the vagal afferents at their origin at the gastro-duodenal mucosa (11). These afferents have glucose-stimulated neurons (12, 13). The motor arm of this reflex involves vagal efferent cholinergic preganglionic neurons synapsing on intragastric NO-containing neurons to mediate gastric relaxation that causes slow gastric emptying (11, 14). Hyperglycemia enhances this reflex by depolarizing the glucose-stimulated afferent neurons by closing K^ATP^ potassium channels (15). Therefore, hyperglycemia causes an increase in gastric compliance and postprandial relaxation (9), a fall in intragastric pressure, reduction in “peristaltic” contractions in the corpus and the antrum, leading to a slow gastric emptying (6). However, there have been reports that hyperglycemia may be associated with fast gastric emptying in experimental animals (16–18). For example, Frank (19) reported that in NIDDM, hyperglycemia causes faster emptying of liquids associated with more significant phasic contractions in the proximal stomach. The site of action of hyperglycemia-induced fast gastric emptying is not known. It was possible that hyperglycemia may act peripherally on the postganglionic/myenteric inhibitory motor neuron of the vagovagal circuit or inhibitory neuromuscular transmission (NMT) to affect fast gastric emptying.

Hyperglycemia has also been reported to induce aberrations in the antral slow waves, such as tachygastria and uncoupling of slow waves, associated with suppression of nitrergic neurotransmission to the ICC that may cause slow gastric emptying (20–22).

Purpose of the present studies was to examine the direct effect of hyperglycemia, distinct from that of other complications of diabetes, on inhibitory NMT. We used a model of endogenous subacute hyperglycemia that distinguishes it from the effect of acute hyperglycemia produced by administration of high glucose and from chronic hyperglycemia that may be associated with long-term complications such as oxidative stress (16). All NOD mice show fast gastric emptying in the first few weeks, but a small number develop slow gastric emptying on a more extended period of follow-up (16) NOD mice develop insulin-dependent diabetes mellitus due to inflammatory destruction of beta cells in the pancreas. In order to determine whether the changes in the NOD mice were due to hyperglycemia and not due to the inflammatory phenotype of the NOD mice were compared any changes in hyperglycemic NOD (H-NOD) mice with those in non-hyperglycemic NOD (NH-NOD) mice.

The inhibitory motor neurons act by releasing not only a nitrergic neurotransmitter but also a purinergic neurotransmitter (2, 3). Therefore, we examined: (1) the effect hyperglycemia on both nitrergic purinergic inhibitory NMT in the gastric antrum; (2) whether the loss of purinergic and nitrergic neurotransmissions was due to loss of neurotransmitter release or a defect in signal transduction; and (3) whether inhibitory NMT was unaffected in NH-NOD mice.

## Materials and Methods

### Ethical

The study protocol was conducted according to the Guide for the Care and Use of Laboratory Animals of the NIH and approved by the Institutional Animal Care and Use Committee of VA Boston Healthcare System (IACUC #357-W-050415 and IACUC#101-W-063012).

### Animals

All animals were obtained from Jackson Laboratory (Bar Harbor, ME, USA). Female C57BL/6J wild-type (WT) mice served as controls. Female NOD mice (NOD/shiLtJ; number 001976) 10-week old were used as models of diabetes. This strain is a widely used model diabetic stomach associated with type-1 diabetes (16). Development of hyperglycemia (diabetes) is more frequently in females NOD mice, and increase with age (23).

NOD mice were tested for glycosuria and blood glucose level. Glycosuria was tested with Clinistix reagent strips for urinalysis (Bayer Corporation, Elkhart, IN, USA) and graded semi-quantitatively. Random blood glucose levels were determined by tail-vein sampling with the ACCU-CHECK complete diabetes-monitoring Kit (model 200, Roche Diagnostics Corporation, Indianapolis, IN, USA). Blood glucose levels below 160 mg/dl were labeled as NH-NOD. Blood glucose levels over 250 mg/dl were labeled H-NOD. When the blood glucose rose above 350 mg/dl, the mice were treated with sub-therapeutic insulin (Humulin 50/50, Eli Lilly and Company, Indianapolis, IN, USA), injected twice daily subcutaneously, to avoid fatality.

### Chemicals

The Krebs solution contained (in mM) NaCl 115.4, KCl 4.6, CaCl_2_⋅2H_2_O 2.5, MgCl_2_⋅2H_2_O 1.2, dextrose 11.5, NaHCO_3_ 21.9 and NaH_2_PO_4_⋅2H_2_O 1.2, and bubbled with a mixture of 5% CO_2_–95% O_2_ (pH 7.4). Apamine, atropine, guanethidine, nifedipine, α,β-methyleneadenosine 5′-triphosphate(α,β-MeATP), Nω-nitro-l-arginine methyl ester hydrochloride, l-NAME hydrochloride, and diethylenetriamine nitric oxide adduct [DETA-NO (DNO)] were obtained from Sigma Chemical (St. Louis, MO, USA). (1R*,2S*)-4-[2-Chloro-6-(methylamino)-9H-purin-9-yl]-2-phosphornooxybicycle[3.1.0]hexane-1-methanol dihydrogen phosphate ester di-ammonium salt (MRS-2279) was obtained from Tocris Biosciences (Ellisville, MO, USA).

### Intracellular Recording

Gastric antrum was removed, and circular muscle strips were prepared. Intracellular recordings of membrane potential were obtained from the smooth muscle cells, using microelectrodes made from glass of 1.2 mm OD × 0.6 mm ID with Omega Dot fiber and filled with 3 M KCl. The resistance of the microelectrodes was between 30 and 80 MΩ as described earlier (3). All membrane potential values were determined by the difference between the stable potential recorded within the cell compared with the balanced zero potential upon withdrawal, as described earlier (2, 3).

### Generation of the pIJP and nIJP

The muscle strips represented nerve-muscle preparations, as they contained myenteric plexus along with innervation of the muscles. Two Ag-AgCl electrodes (0.26 mm diameter) positioned above and below the preparation perpendicular to its longitudinal axis, and 5 mm away from the recording microelectrode was used to deliver trans-mural stimulations. These electrodes were insulated up to 2 mm from their tips and connected to a stimulator (Grass S-88) in series with a stimulus isolation unit (Grass SIU5). Optimal stimulus parameters (70 V, 0.7–1 ms duration square pulses at 20 Hz for 0.5 s) were used. The perfusing solution contained atropine (1 µM) and guanethidine (5 µM) to create non-adrenergic, non-cholinergic (NANC) conditions. The perfusing solution also included nifedipine (0.1 µM) that reduced contraction of the muscle. We examined the IJPs in during the period when slow waves were absent. The compound inhibitory junction potentials (cIJPs) in the stomach can be resolved into a large amplitude fast purinergic IJP (pIJP) and a small amplitude slow nitrergic IJP (nIJP) components. We used perfusion with apamin (0.3 µM) or MRS-2279 (5 µM) to block the pIJP and l-nitro-l-arginine methyl ester (200 µM) to block the nIJP.

### Statistical Analysis

All statistical evaluations were performed with Statistics software SPSS 21.0 (IBM). The data were expressed as the mean ± SD. For comparison between two data sets, a Student’s *t*-test was used. Analysis of differences between multiple groups of data was performed with one-way ANOVA followed by a *post hoc* Bonferroni test. Differences were considered be significant at a *P*-value less than 0.05.

## Results

### Studies in NOD Mice Background Data

We divided the NOD mice into H-NOD mice and NH-NOD mice groups. C57BL/6J mice with matching age to the two groups were used as WT controls. Sex, age, and weight of the NH-NOD mice and matching WT-1; and H-NOD mice and matching WT-2 are summarized in Table 1. Mice in all groups were females. Their weights were comparable except that the H-NOD mice had significantly lower weight than the other groups.

**Table 1:** Background data.

	WT1	NH-NOD	WT2	H-NOD
*N*	10	10	10	10
Sex	F	F	F	F
Age (week)	14.4 ± 0.7 (12–25)	14.6 ± 0.5 (12–25)	26.8 ± 5.5 (13–47)	27.0 ± 5.5 (13–47)
Weight (g)	21.0 ± 0.7 (19–28)	21.8 ± 0.6 (20–28)	24.2 ± 1.1 (23–34)	16.4 ± 0.5* (15–18)
Blood sugar (mg/dl)	155.4 ± 2.0 (135–159)	158.6 ± 1.2 (151–159)	156.6 ± 2.0 (148–159)	284.6 ± 3.3** (255–390)

*P < 0.001.

**P < 0.001.

In the WT-1 controls for NH-NOD and the NH-NOD mice, random blood sugar levels were 155.4 ± 2.0 mg/dl (135–159 mg/dl, *n* = 11) and 162.6 ± 1.2 mg/dl (160–166 mg/dl, *n* = 11). This difference was not significant (*P* = 0.09). In the WT-2 controls for H-NOD, random blood sugar levels were 155.6 ± 2.0 mg/dl (148–159 mg/dl, *n* = 11) and in the H-NOD mice, random blood sugar levels were 284.6 ± 3.2 mg/dl (255–390 mg/dl, *n* = 11). These values are significantly higher than their controls (*P* < 0.0001). Blood sugar levels in H-NOD mice were also significantly greater than in NH-NOD mice (*P* < 0.001).

### Resting Membrane Potential (RMP)

In the NH-NOD mice, RMP recorded from the smooth muscle cells was −51.7 ± 0.5 mV a value that was not different from that in the WT1 controls −51.5 ± 0.63 mV (*P* > 0.5, *n* = 18 cells in 10 animals). In the H-NOD mice, the RMP was −48.4 ± 0.4 mV; these values are significantly lower than their WT2 controls −55.0 ± 1.9 mV (*P* < 0.001, *n* = 18 cells in 10 animals) and the NH-NOD mice (*P* < 0.0001).

### Compound IJP

**Figure 1:**
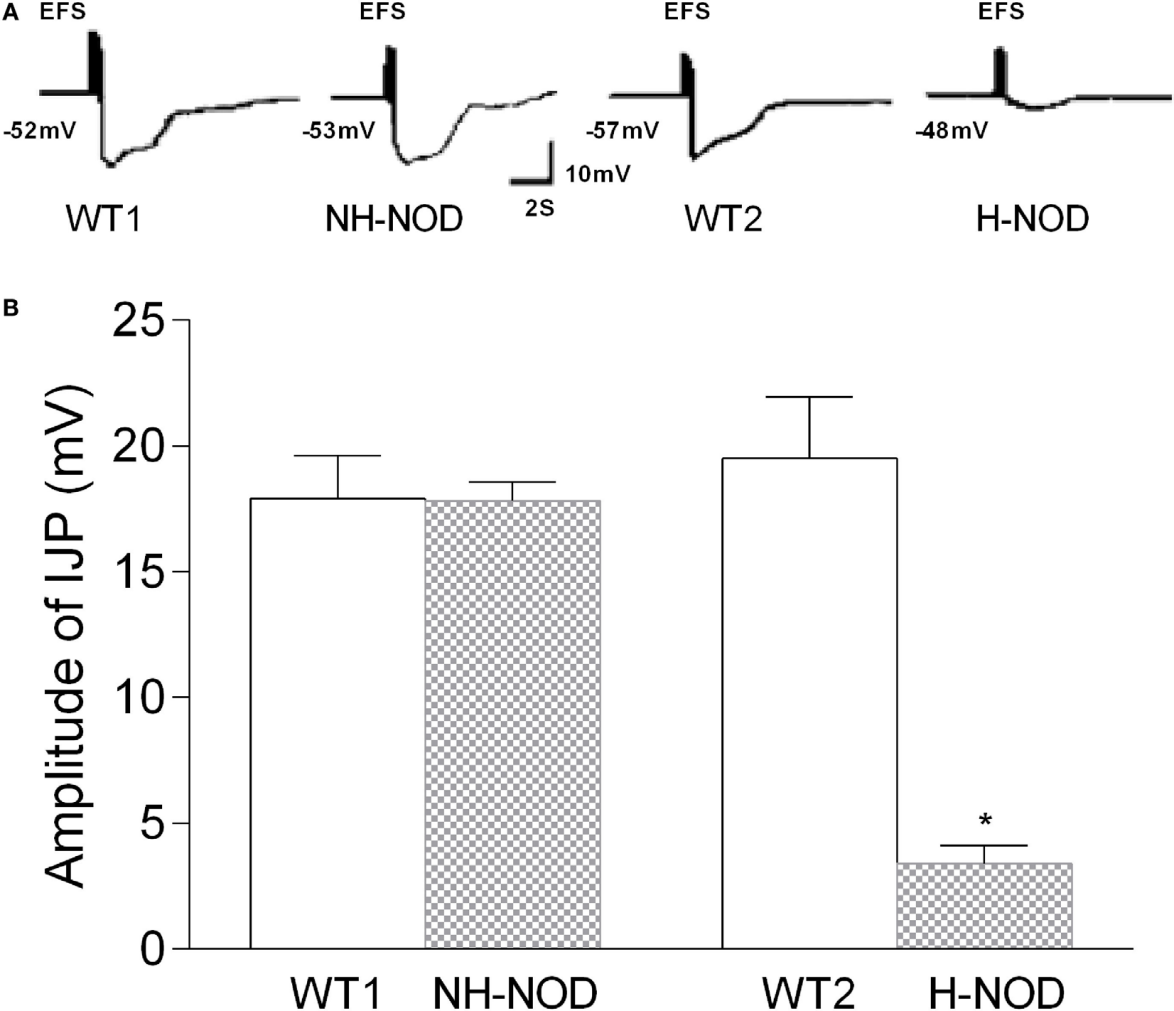
Compound IJP in wild-type (WT) controls, non-hyperglycemic NOD (NH-NOD), and hyperglycemic NOD (H-NOD). Panel **(A)** shows actual examples and panel **(B)** shows cumulative data on the amplitudes of the compound IJP. Note that in WT controls, the compound IJP consist of overlapping fast and slow IJPs. The amplitude of the compound IJP is due to the fast IJP [peaking at around 1 s after the onset of electric field stimulus (EFS)]. In NH-NOD mice amplitude of the compound IJP was not affected. However, but in H-NOD the compound IJP was markedly suppressed, and that peaked at around 4 s after the onset of the EFS, suggesting that fast IJP may be obliterated the slow IJP suppressed. Values are mean ± SD. *P < 0.001.

Under the NANC condition, the cIJP consists of two overlapping components, fast and slow IJPs. The fast IJP is large in amplitude and peaks at around 1 s, and the slow IJP is less than half the magnitude of the of the fast IJP peaks at around 4 s after the onset of the stimulus. The fast IJP provide the maximal amplitude of the compound IJP in the stomach (Figure 1). The amplitude of the compound IJP was 17.9 ± 0.5 mV in the WT-1 controls and NH-NOD it was 17.8 ± 0.2 mV (*P* = 0.8). In WT-2 control animals, the magnitude of the compound IJP was 19.5 ± 0.5 mV, and in H-NOD it was 3.4 ± 0.2 mV (*P* < 0.001). Interestingly, the peak amplitude of the compound IJP in WT-2 controls occurred at 1 s after the onset of the stimulus, but the peak amplitude in H-NOD took place at around 4 s after the start of the stimulus. These observations suggested that the peak amplitude of the compound IJP reflect the fast IJP and the peak amplitude of the reduced compound IJP in H-NOD indicated nearly lost fast IJP but suppressed slow IJP. The fast IJP is purinergic, and the slow IJP is nitrergic. Therefore, further studies were performed on chemically isolated pIJP and nIJPs.

The fast IJP of the compound IJP was blocked by apamin (0.3 µM), or by a selective inhibitor of P2y1 receptors, MRS-2279 (5 µM) (24). On the other hand, the slow component was suppressed by l-NAME, an inhibitor of NOS and represented nIJP. The potency order of the P2y1 antagonists is MRS-2500 > MRS-2279 > MRS-2179 (25). However, pIJP was fully revealed after treatment with l-NAME and nIJP was fully revealed after treatment with apamin or MRS-2279.

### Purinergic IJP

**Figure 2:**
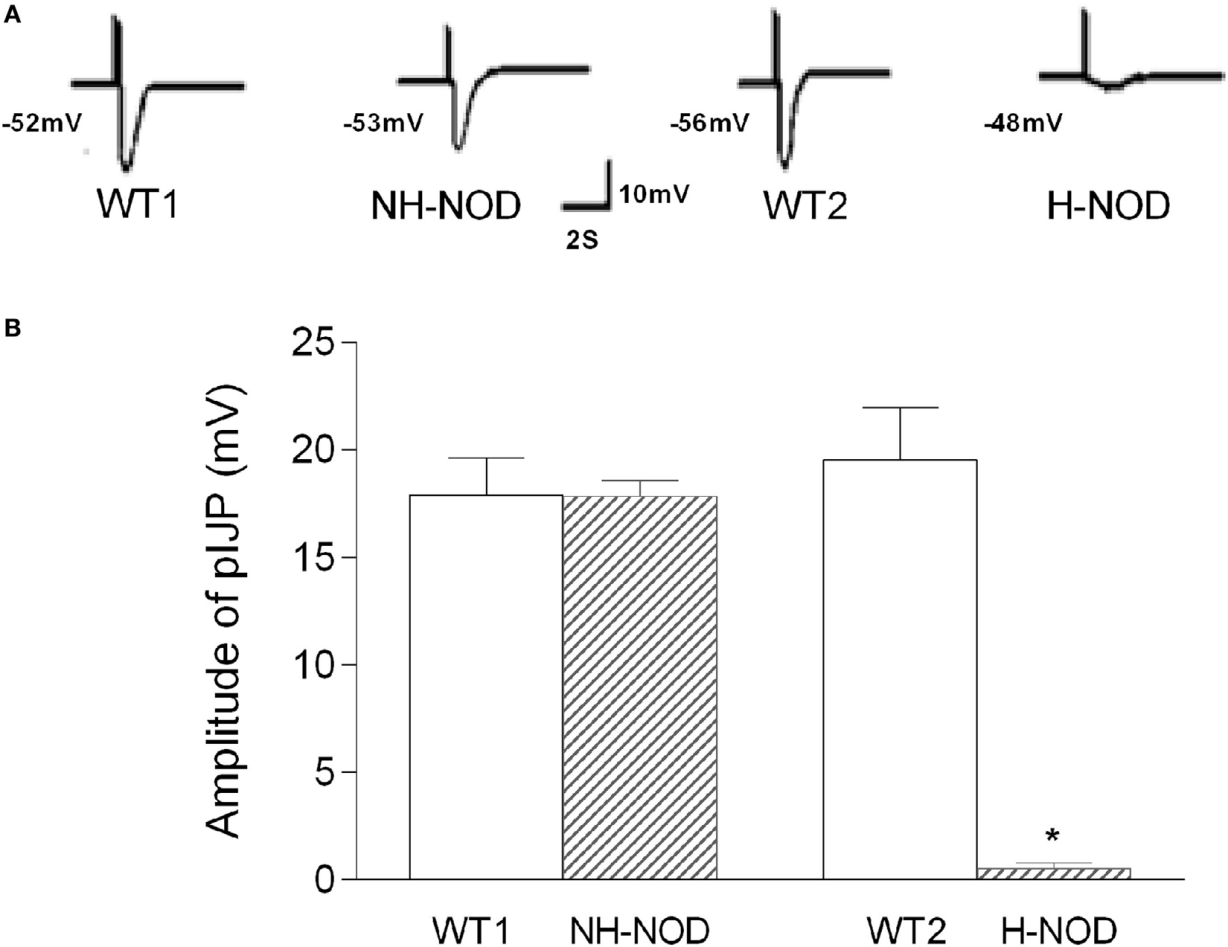
Purinergic IJP (pIJP) in wild-type (WT) controls, non-hyperglycemic NOD, and hyperglycemic NOD (H-NOD). Panel **(A)** shows actual examples and panel **(B)** shows cumulative data on the amplitudes of the pIJP. Note that the pIJP was unaltered as compared to the WT control but almost abolished in H-NOD mice. Values are mean ± SD. *P < 0.0001.

The pIJP in NH-NOD and H-NOD and their respective controls are shown in Figure 2. The pIJP in the NH-NOD mice was 17.8 ± 0.2 mV a value that was not different from 17.9 ± 0.5 mV in the WT-1 control (*P* > 0.05, *n* = 13 cells in 10 animals). In the H-NOD mice, pIJP was significantly reduced (97%) compared with WT-2 controls (0.5 ± 0.1 vs. 19.5 ± 0.6 mV; *P* < 0.0001, *n* = 18 cells in 10 animals), or compared with the value in NH-NOD mice 17.8 ± 0.2 mV (*P* < 0.0001).

### Hyperpolarization to α,β-MeATP

**Figure 3:**
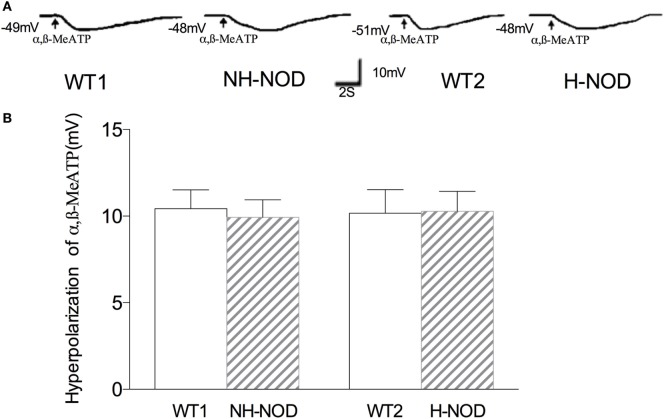
Membrane hyperpolarization of purinergic agonist α,β-MeATP. Panel **(A)** shows actual examples and panel **(B)** shows cumulative data on the amplitudes of the purinergic inhibitory junction potential. Note that hyperpolarization to α,β-MeATP was no different in hyperglycemic NOD as compared to wild-type (WT) control.

To determine changes in responsiveness of the postjunctional apparatus to the purinergic neurotransmitter, we examined smooth muscle responses to purinergic receptor agonist α,β-MeATP (26). Figure 3 shows membrane potential responses to exogenous α,β-MeATP in H-NOD, NH-NOD, and their respective controls. Exogenous α,β-MeATP caused hyperpolarization of 10.2 ± 0.3 mV (*n* = 12 cells in 10 animals) in H-NOD, which has no difference compared with 10.2 ± 0.4 mV in WT2 controls (17 cells from 10 animals; *P* > 0.5). In the NH-NOD mice, membrane hyperpolarization in α,β-MeATP (33 µM) was 9.9 ± 0.4 mV, a value very close to that in the WT1 10.4 ± 0.3 mV (*P* > 0.05, 13 cells from 10 animals). Overall, these results suggest that the suppression of purinergic responses in the diabetic stomach is not due suppressed purinergic signal transduction in PDGFRα or smooth muscle cells (27).

### Nitrergic IJP

**Figure 4:**
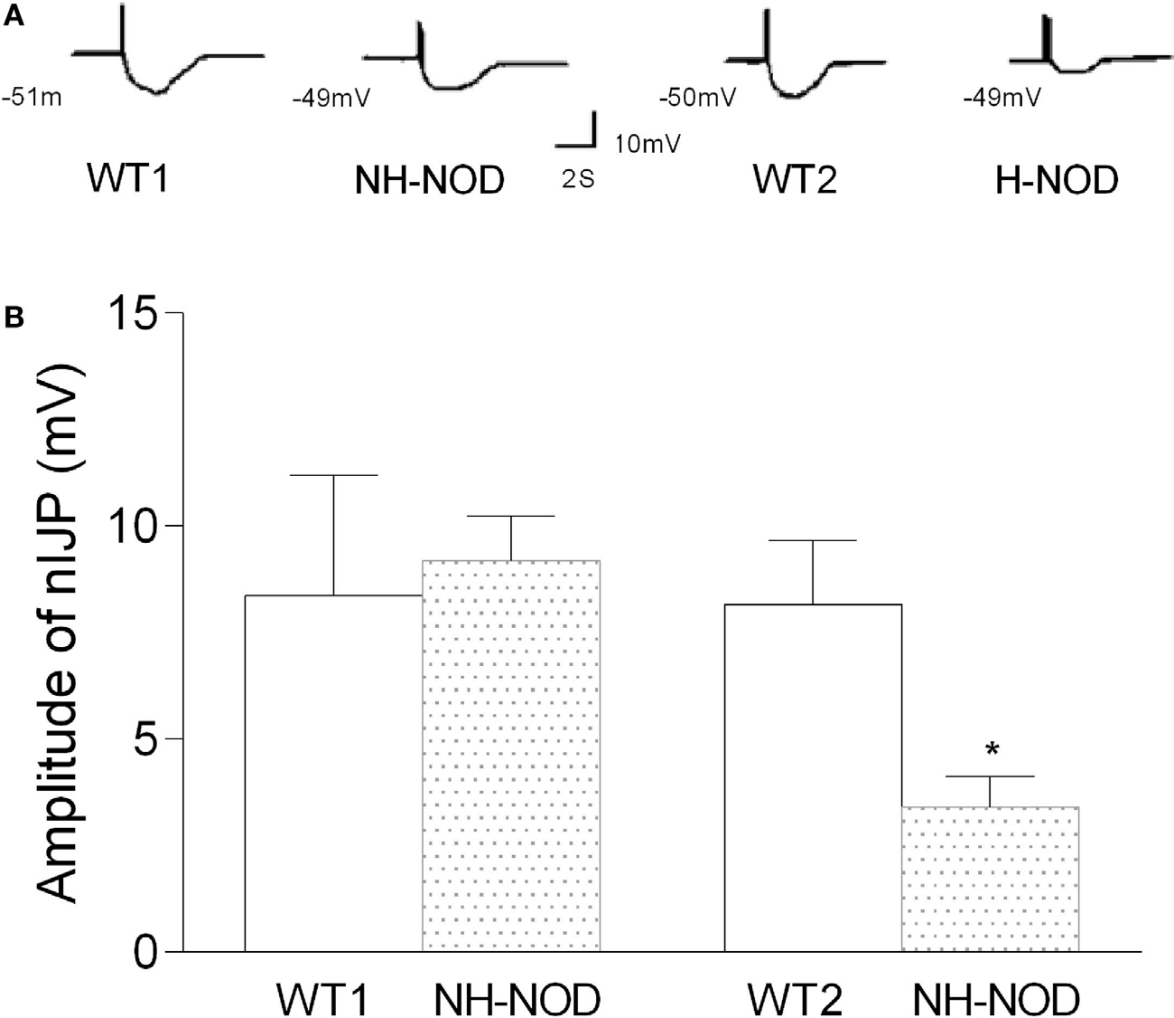
Nitrergic IJP (nIJP) in wild-type (WT) controls, non-hyperglycemic NOD (NH-NOD), and hyperglycemic NOD. Panel **(A)** shows actual examples and panel **(B)** shows cumulative data on the amplitudes of the nIJP. Note that in NH-NOD the nIJP was reduced to 40% of WT controls. Values are mean ± SD. *P < 0.0001.

The nIJP was isolated by performing studies under NANC conditions and by blocking purinergic responses with P2y1 receptor inhibitors MRS-2279. Figure 4 shows changes of nIJP in each group. In NH-NOD mice, nIJP was 9.2 ± 0.3 mV, value not different from 8.4 ± 0.8 mV in WT-1 controls (*P* > 0.05, *n* = 11 cells in 10 animals) showing that NH-NOD mice have no impaired nitrergic NMT. In the H-NOD mice nIJP was significantly reduced by 58% as compared to that in their WT controls (3.4 ± 0.2 mV in H-NOD compared with 8.2 ± 0.3 mV in WT-2 controls; *P* < 0.0001, *n* = 18 cells in 10 animals); and as compared the NH-NOD (*P* < 0.0001).

### Hyperpolarization to DNO

**Figure 5:**
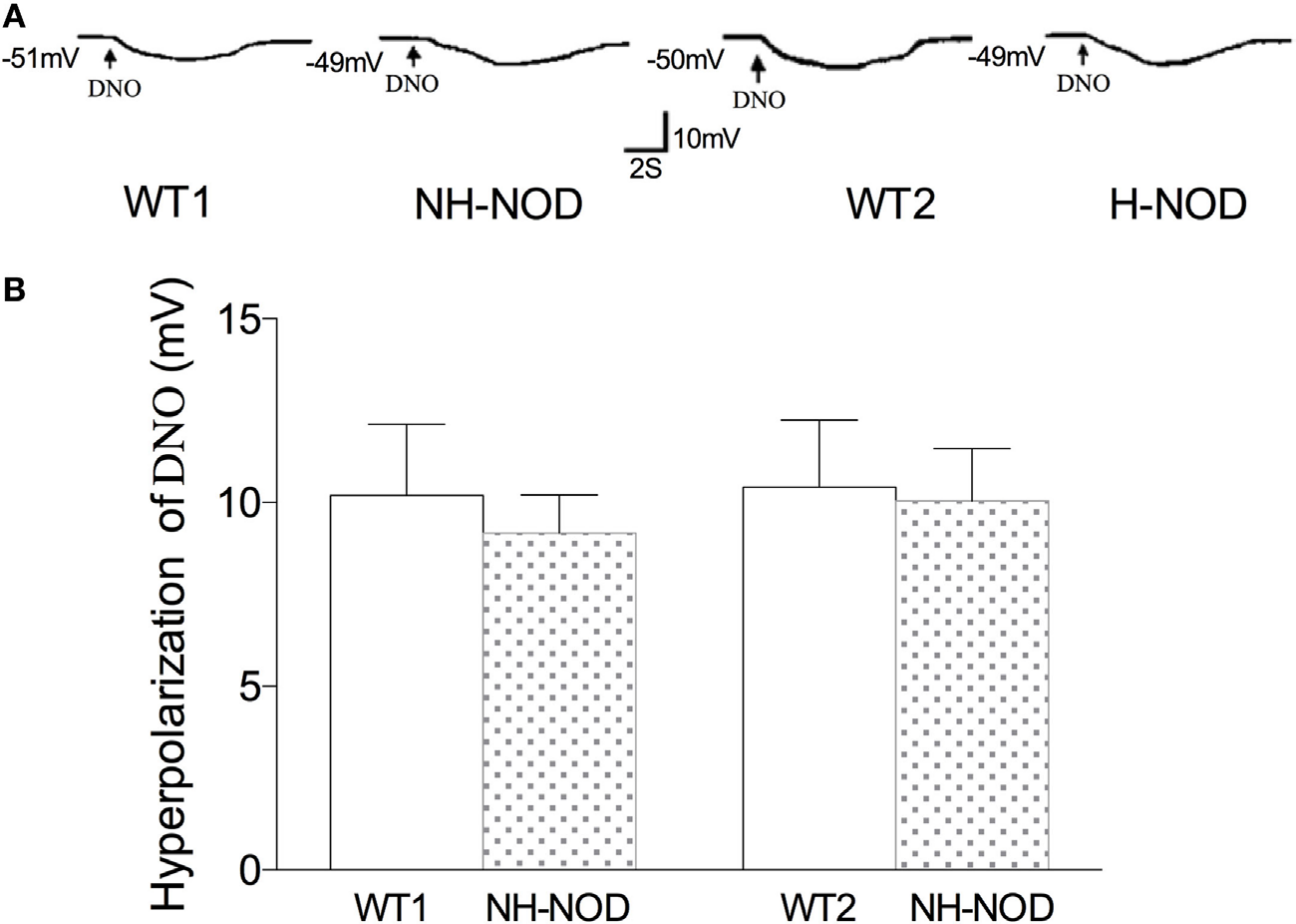
Membrane hyperpolarization to nitrergic agonist, diethylenetriamine nitric oxide adduct (DNO). Panel **(A)** shows actual examples and panel **(B)** shows cumulative data on the amplitudes of the nitrergic inhibitory junction potential. Note that hyperpolarization to DNO in hyperglycemic NOD (H-NOD) was similar to wild-type (WT) control.

To determine the responsiveness of the postjunctional apparatus to the nitrergic neurotransmitter, we examined smooth muscle responses to nitrergic receptor agonist, DNO, a direct NO donor that interact with heme moiety of its intracellular receptor GTP-cyclase (28). As shown in Figure 5, in the WT-1 control, DNO (660 µM) produced membrane hyperpolarization of 10.2 ± 0.5 mV, and it was 9.2 ± 0.6 mV in the NH-NOD mice. This difference was not significant (*P* > 0.05, *n* = 15 cells in 10 animals). In the H-NOD mice, membrane hyperpolarization evoked by DNO was 10.0 ± 0.4 mV that was not different from that in WT-2 controls 10.4 ± 0.5 mV (*P* > 0.05, *n* = 12 cells in 10 animals). Moreover, the values of DNO-induced hyperpolarization in NH-NOD 9.2 ± 0.6 mV and H-NOD 10.0 ± 0.4 mV were not significantly different (*P* > 0.5). These observations show that postjunctional signal transduction evoked by DNO was not affected in NH-NOD or H-NOD mice.

## Discussion

Fast gastric emptying is now increasingly recognized as a complication of early diabetes and hyperglycemia in animal models of diabetes including NOD mice (16), lepr^db/db^ mice (18) and streptozotocin-induced diabetes mellitus (17). Clinically also, diabetic patients with fast gastric emptying are being recognized with higher frequency even in well-established chronic diabetics. In one study of diabetic patients who underwent gastric emptying studies for clinical symptoms, fast gastric emptying was found to be fast in 22%, normal in 42%; and slow in 36% of the cases (29).

These studies show that inhibitory NMT, involving both purinergic and nitrergic neurotransmissions are suppressed in the gastric antrum during early stages of development of hyperglycemia in the NOD mice, suggesting that subacute hyperglycemia is the likely cause of these changes. Moreover, the effect of hyperglycemia was due to reduced transmitter release and not due to impaired signal transduction in the interstitial cells or smooth muscles. By suppressing inhibitory NMT in the gastric antrum, hyperglycemia may lead to fast gastric emptying.

An unexpected finding of our study was that purinergic neurotransmission was severely impaired by hyperglycemia. It is assumed that the purinergic NMT is preserved in the diabetic stomach because PDGFRα positive fibroblasts are preserved in the diabetic stomach (27). However, we found that the action of the purinergic neurotransmitter mimic was preserved hyperglycemia, suggesting that the loss of purinergic NMT in hyperglycemia was due to impaired neurotransmitter release. The importance of the loss of purinergic NMT is currently unknown (30). Studies in P2Y1 knock out mice may help define the role of purinergic NMT is gastric emptying (31).

The present study shows that nIJP is also impaired in the gastric antrum of subacutely H-NOD mice. The nIJP was reduced by almost 60% as compared with that of WT mice. Surprisingly, however, this reduction was less severe than that of the pIJP, which was nearly abolished. Impairment of nitrergic neurotransmission in acute or subacute hyperglycemia has not been reported before. Impaired nitrergic NMT of hyperglycemia may be due to impaired release of NO or impaired signal transduction of normally released NO or defective neural release of NO. There is a view that ICC-IM is required to transduce nitrergic neural signals to smooth muscles. However, this view has been questioned (32, 33). Moreover, any role for ICC in suppressing nitrergic NMT in hyperglycemia is unlikely because in acute or subacute hyperglycemia ICC are either not affected (34) or may even be increased (18). Moreover, the current studies show that DNO induced hyperpolarization was not impaired in subacutely H-NOD mice. All these observations suggest that defective inhibitory neurotransmission in the diabetic stomach was due to reduced transmitter release.

Hyperglycemia has been reported act on different targets to enhance gastric motility. It has been reported that hyperglycemia may increase ghrelin release that can enhance gastric emptying (35). Hyperglycemia has also been reported to suppress the vagovagal reflex that is activated by cholecystokinin and secretin (12, 36). The motor pathway of this reflex involves the release of peptide VIP to cause gastric relaxation. Hyperglycemia antagonizes this reflex by hyperpolarizing the glucose-inhibited neurons in the afferent pathway by closing TWIK-related spinal cord potassium channels (36), resulting in antagonism of gastric relaxation that may lead to fast gastric emptying by hyperglycemia.

It has also been reported that hyperglycemic Lepr^db/db^ mice with fast gastric emptying were reported to exhibit increased sensitivity to carbachol in causing contractions of the antral smooth muscle. The increased sensitivity of the smooth muscle was thought to be due to gain in the number of ICC in this model (18). However, increased excitability of smooth muscles is a well-established phenomenon in hyperglycemia (37). Consistent with the increased excitability of antral smooth muscle in hyperglycemia, we found that smooth muscles were depolarized in H-NOD mice. Loss of inhibitory NMT may contribute to the smooth muscle excitability (37). Loss of inhibitory NMT and increased excitability of gastric fundus, corpus and antrum may lead to loss of compliance and enhanced contractions and accelerate gastric emptying. On the other hand, loss on the nitrergic inhibitory transmission is also known to cause delayed gastric emptying by causing loss of relaxation of the pyloric sphincter (38, 39). Further studies are needed to resolve this important paradox.

Hyperglycemia has been reported to stimulate mechanosensitive vagal afferents of the vagovagal reflex that cause gastric relaxation and delayed gastric emptying by releasing inhibitory neurons at the neuromuscular junction (11, 15). However, as shown in the present study, hyperglycemia can act directly on the inhibitory motor neurons that are final mediators of the gastric inhibitory vagovagal circuits to suppress inhibitory NMT. Suppression of inhibitory NMT directly would nullify any stimulatory action of hyperglycemia on the afferent neurons that are upstream on the vagovagal circuit.

The mechanism of suppression of inhibitory neurotransmitter release by hyperglycemia would require further studies. However, myosin-5a (Myo5a) dysfunction may be involved in hyperglycemia-induced suppression of the transmitter release. Subacute endogenous hyperglycemia in NOD mice is due to insulin deficiency. Insulin deficiency has been shown to cause the reduced function of AKT serine/threonine kinase 2 that suppresses the function of the intracellular motor, Myo5a. Reduced function of Myo5a suppresses membrane translocation of glucose transporter type 4 that reduces cellular glucose uptake (40). Reduced PI3/Akt signaling is seen in the diabetic stomach that is reversed by the glial cell-derived neurotrophic factor (41). It has been shown that Myo5a deficiency also causes loss of transport of the purinergic secretory vesicles and the enzyme nNOSα from the interior of varicosity to the plasma membrane where neurotransmitter release occurs and cause loss of neurotransmission (42–44). Moreover, Myo5a is reportedly reduced in nerves in the jejunum of diabetic rats (45). These observations suggest that Myo5a dysfunction due to insulin deficiency could be a common underlying factor in impaired purinergic and nitrergic NMT in H-NOD mice.

## Ethics Statement

The study protocol was conducted according to the Guide for the Care and Use of Laboratory Animals of the NIH and approved by the Institutional Animal Care and Use Committee of VA Boston Healthcare System (IACUC #357-W-050415 and IACUC#101-W-063012).

## Author Contributions

X-DH: performed the experiments and analyzed the data; Y-MG: analyzed the data and wrote the article; RG: conceived and designed the experiments and wrote the article.

## Conflict of Interest Statement

The authors declare that the research was conducted in the absence of any commercial or financial relationships that could be construed as a potential conflict of interest.
